# Occasional Latency of the Symptoms in Advanced Carcinoma Uteri

**Published:** 1853-08

**Authors:** 


*Occasional Latency of the Symptoms in advanced Carcinoma Uteri.*—In the earlier stages of cancer of the uterus, the disease is, as a general rule, accompanied by few, or indeed no, well-marked dynamic symptoms. Patients themselves, and sometimes also the members of the profession, seem to expect that the advent and presence of this fatal malady should be very constantly accompanied with local pain and suffering. The reverse, however, of all this seems to be the general rule. In fact, it rarely happens that a patient affected with uterine cancer applies at all for medical advice till the disease has advanced beyond the stage of deposit, and has already made more or less progress in the stage of ulceration. Even then the local symptoms which excite the patient's attention are usually not the expected pathognomic pain, but occasional attacks of hemorrhage, attended with leucorrhœal discharge. Or, if the pain is present, it often, as yet only amounts to a sensation of discomfort and uneasiness, and not to a feeling of actual suffering. Nay, sometimes any feeling of pain in the uterus or uterine region itself never supervenes at all, or not till the very last period of the affection. In the course of practice, I have happened to see a number of cases to which this remark applies. Instances also occasionally occur where the patient suffers more or less severely from pain; but that symptom is in the form of a sympathetic or reflex pain, situated, not in the uterus, but in the limbs, loins, or some other distant part. Several years ago, I had occasion to examine a case in which the cervix uteri was entirely eaten away by extensive cancerous ulceration; but without any marked local pain. The patient, however, had complained so much of pain in the mamma, that local anodyne and other applications had been applied to that part of the body. Dr. Davidson told me the particulars of a case, in which the patient complained to her medical attendant of nothing during life, except a series of severe urinary symptoms, for which she had ineffectually undergone a variety of treatment. On opening her body after death, the coats of the bladder were found deeply implicated in a mass of ulcerated uterine carcinoma. The following case, which I saw within the last few weeks, with Dr. Cowan, in a patient who came from a distance in the country, is one of the most striking illustrations which I have met with of the occasional latency of the local symptoms of cancer of the uterus, even in a very advanced and ulcerated stage, and of the transference, as it were, of the principal suffering and symptoms to another organ:—

Case.—A lady, æt. 43, married at a very early age, and the mother of six children, had enjoyed the most robust health until twelve months ago. About that period, she first observed a white discharge from the vagina, which she believed to be common leucorrhœa. There likewise occurred repeated discharges of blood: sometimes in large coagulated masses and shreds. At the same time, the catamenia recurred with regularity, and without pain. About three months since, she first complained of such prostration as prevented her taking her usual amount of exercise. Difficulty and pain also in passing water, and latterly incontinence of urine supervened. During all this period, she experienced no feeling of uneasiness referable to the uterus itself; nor were the leucorrhœa or menorrhagia of a nature or extent calculated to excite in the mind of the patient any feelings of alarm. In fact, the principal, and, according to her own account, her almost sole symptoms were the debility already mentioned, and the painful dysuria, which had, however, been relieved by alkalies.

On making a vaginal examination, I found the cervix uteri, with the upper and anterior part of the vagina, the seat of extensive carcinomatous induration and ulceration. The disease in its ulcerative process had, in fact, proceeded so far at one point that it had implicated and *already perforated* the neck of the bladder,—thus leading first to the dysuria, and subsequently to the incontinence of urine, of which the patient so much complained.

In a note from Dr. Cowan, dated December 20th, he states:—"At present our patient's appetite is good; bowels regular. She sleeps well, and the general appearance is improved, rather than otherwise, since you saw her. All she complains of is, general debility, incontinence of urine, with a thin, white non-acrid discharge, and occasionally (but not constantly) heat in the region of the uterus, unaccompanied with pain. All other symptoms of extensive uterine disease are absent."—*Dr. Simpson's Contributions to Obstetric Pathology and Practice.*

